# Inorganic Nanomaterials Meet the Immune System: An Intricate Balance

**DOI:** 10.1002/adhm.202404795

**Published:** 2025-03-13

**Authors:** Gloria Pizzoli, Marco Gargaro, Giuliana Drava, Valerio Voliani

**Affiliations:** ^1^ Department of Pharmacy School of Medical and Pharmaceutical Sciences University of Genoa Viale Cembrano 4 Genoa 16148 Italy; ^2^ Center for Nanotechnology Innovation @NEST Istituto Italiano di Tecnologia Piazza San Silvestro 12 Pisa 56127 Italy; ^3^ Department of Pharmaceutical Sciences University of Perugia Via del Giochetto 1 Perugia 06126 Italy

**Keywords:** copper, gold, immune system, nanomaterials, oncology, silver

## Abstract

The immune system provides defense against foreign agents that are considered harmful for the organism. Inorganic nanomaterials can be recognized by the immune system as antigens, inducing an immune reaction dependent on the patient's immunological anamnesis and from several factors including size, shape, and the chemical nature of the nanoparticles. Furthermore, nanomaterials‐driven immunomodulation might be exploited for therapeutic purposes, opening new horizons in oncology and beyond. In this scenario, we present a critical review of the state of the art regarding the preclinical evaluation of the effects of the most promising metals for biomedical applications (gold, silver, and copper) on the immune system. Because exploiting the interactions between the immune system and inorganic nanomaterials may result in a game changer for the management of (non)communicable diseases, within this review we encounter the need to summarize and organize the plethora of sometimes inconsistent information, analyzing the challenges and providing the expected perspectives. The field is still in its infancy, and our work emphasizes that a deep understanding on the influence of the features of metal nanomaterials on the immune system in both cultured cells and animal models is pivotal for the safe translation of nanotherapeutics to the clinical practice.

## Graphical abstract

Metal nanomaterials influence the immune responses, such as cytokine production and T‐cell activity. Unintended immune modulation can have significant consequences while its fine‐tuning may transform the oncology care. Within this review, a critical analysis is offered regarding the nano/immune‐interaction and suggests the future research directions to support the safe clinical translation of inorganic nanomaterials.

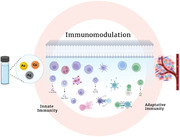

## 1 Introduction

**Figure 1:**
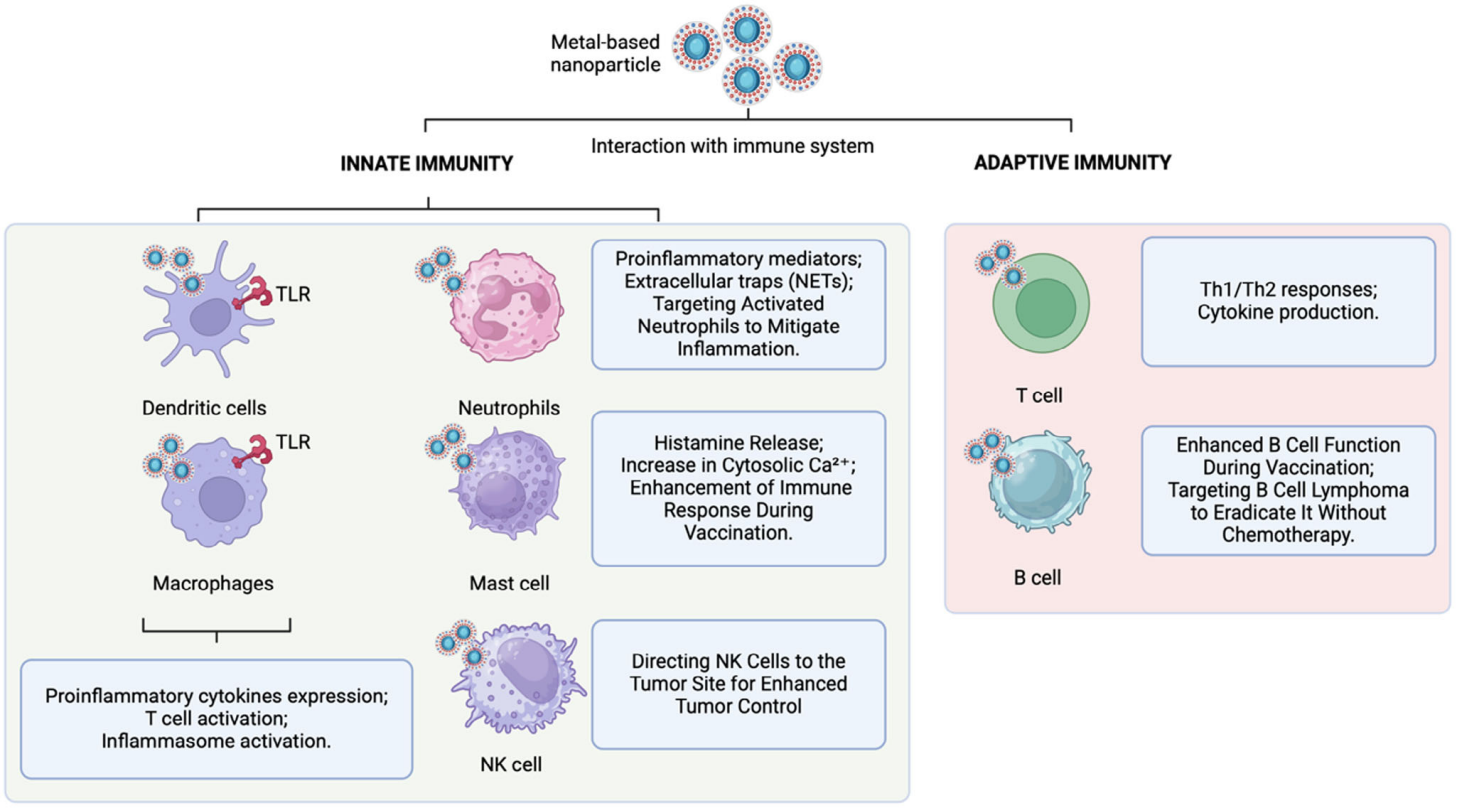
Summary of the potential interactions of noble metal nanoparticles with the immune system. Rearranged with permission.^[^ ^22^ ^]^ Copyright© 2015, Yueh‐Hsia Luo et al.

Several daily products contain nanomaterials, but the killing application is still in the medical field.^[^ ^1^ ^]^ In this regard, a growing excitement is associated with the medical and economic impact that nano‐technological approaches may have on the healthcare system, despite some undue pessimism that is sometimes recognized, such as in the history of cancer chemotherapy.^[^ ^1^, ^2^ ^]^ The very first nanotherapeutic was introduced in human therapy at the end of the last century to increase the efficacy of a poorly bioavailable drug (Doxil®, liposomes loaded with doxorubicin).^[^ ^3^ ^]^ Actually, there are ≈60 nanotherapeutics in the market, generally organic nanomaterials designed as drug delivery platforms.^[^ ^4^, ^5^, ^6^ ^]^ Indeed, it should be noted that despite the noble metal nanomaterials having the potential to shift the current treatment paradigms in oncology due to the peculiar physical and physiological features, they are still at the bench‐side.^[^ ^7^, ^8^ ^]^ The pathway for translating metal nanomaterials to the clinical practice is generally challenging for several reasons, especially regarding the safety.^[^ ^7^, ^9^ ^]^ Obviously, a treatment should be as effective as possible with the best sparing effect on healthy tissues and in general on the whole organism.^[^ ^10^ ^]^ Among the most prominent issues, the persistence after the action was one of the most problematic for metal nanotherapeutics translation, and it has been recently addressed with the introduction of the ultrasmall‐in‐nano approach for the design of disassembling architectures.^[^ ^11^, ^12^, ^13^, ^14^ ^]^ On the other hand, the first bio‐interaction that nanomaterials experience after administration is with the immune system.^[^ ^15^ ^]^ The immune system is a complex biological organization that defends against foreign agents considered harmful to the organism.^[^ ^16^ ^]^ It is often divided into innate and adaptive (**Figure** **1**). The first is highly reactive against any foreign intrusion, and the latter is less responsive but provides memory of the intrusion to respond to following potential interactions with similar antigens.^[^ ^15^ ^]^ Interestingly, a wide number of studies reported that inorganic nanomaterials can interact with both the innate and adaptive immunity (Figure 1).^[^ ^17^ ^]^ After the administration, the first interacting cells are usually the phagocytic ones.^[^ ^18^ ^]^ The phagocytic cells are bone marrow‐derived cells of myeloid origin responsible for controlling the initial response to infection.^[^ ^19^ ^]^ Monocytes, macrophages, neutrophils, dendritic cells (DCs), osteoclasts, and eosinophils are considered professional phagocytes, and they are involved in the uptake and destruction of invading pathogens as well as in tissue homeostasis.^[^ ^20^, ^21^ ^]^

The interactions of nanomaterials with the immune system are still not entirely known and can trigger inflammations and/or immune‐stimulation/suppression. Thus, exploring the nano/immune‐interaction will shed light on one of the main concerns regarding metal nanotherapeutics translation.^[^ ^5^ ^]^ It should be noted that nanomaterials‐driven immunomodulation may also open new horizons in healthcare. Indeed, immunomodulation is not necessarily an adverse effect leading to toxicity, yet it can be exploited for therapeutic purposes because, in principle, the immune system can be stimulated for self‐healing.^[^ ^23^ ^]^ For instance, the mechanism through which vaccines protect the body from common diseases is the administration of agents that resemble a specific disease‐inducing pathogen to stimulate the immune cell to recognize it (memory effect).^[^ ^24^ ^]^ The immune responses may not be triggered solely by the chemical composition of the nanomaterials, yet several factors including size, shape, and the patient immunological anamnesis can play an essential role in driving the immune response. It is still debated whether the immune system can recognize inorganic compounds as epitopes and whether specific antibodies against these materials are produced. Moreover, inorganic nanomaterials can acquire a new immunological identity once in the bloodstream, owing to protein corona formation. Thus, the immunomodulation may be an equilibrium between the chemical nature of the nanomaterials, the external decoration, and opsonization. In the literature, inconsistent results regarding nano/immune interaction are present because of the complex interdependence of several variables and the lack of standardized experimental procedures. Thus, within this review, we have systematically analyzed the most prominent findings regarding the immunomodulation associated with metal nanomaterials including gold, silver, and copper, highlighting the dependence from the surface decoration, the size, and the chemical nature of the metal. Beyond an organic summary of the knowledge regarding the nano/immune‐interaction, we provide the perspectives and the expected impact that inorganic nanomaterials will have on relevant medical applications if their translation to clinical oncology will be supported.

## 2 The Immune System and its Role in Oncology

The immune system plays a central role in oncology, serving as a barrier against tumor development and, paradoxically, as a promoter of cancer progression. Immune surveillance mechanisms identify and eliminate emerging malignant cells; however, tumors can often evade detection by exploiting immune regulatory pathways, which leads to their growth and metastasis.^[^ ^25^ ^]^ This duality underscores the complexity of targeting immune responses for therapeutic benefits, as immune mechanisms that typically protect against cancer can also be co‐opted to support tumor survival under certain conditions.

**Figure 2:**
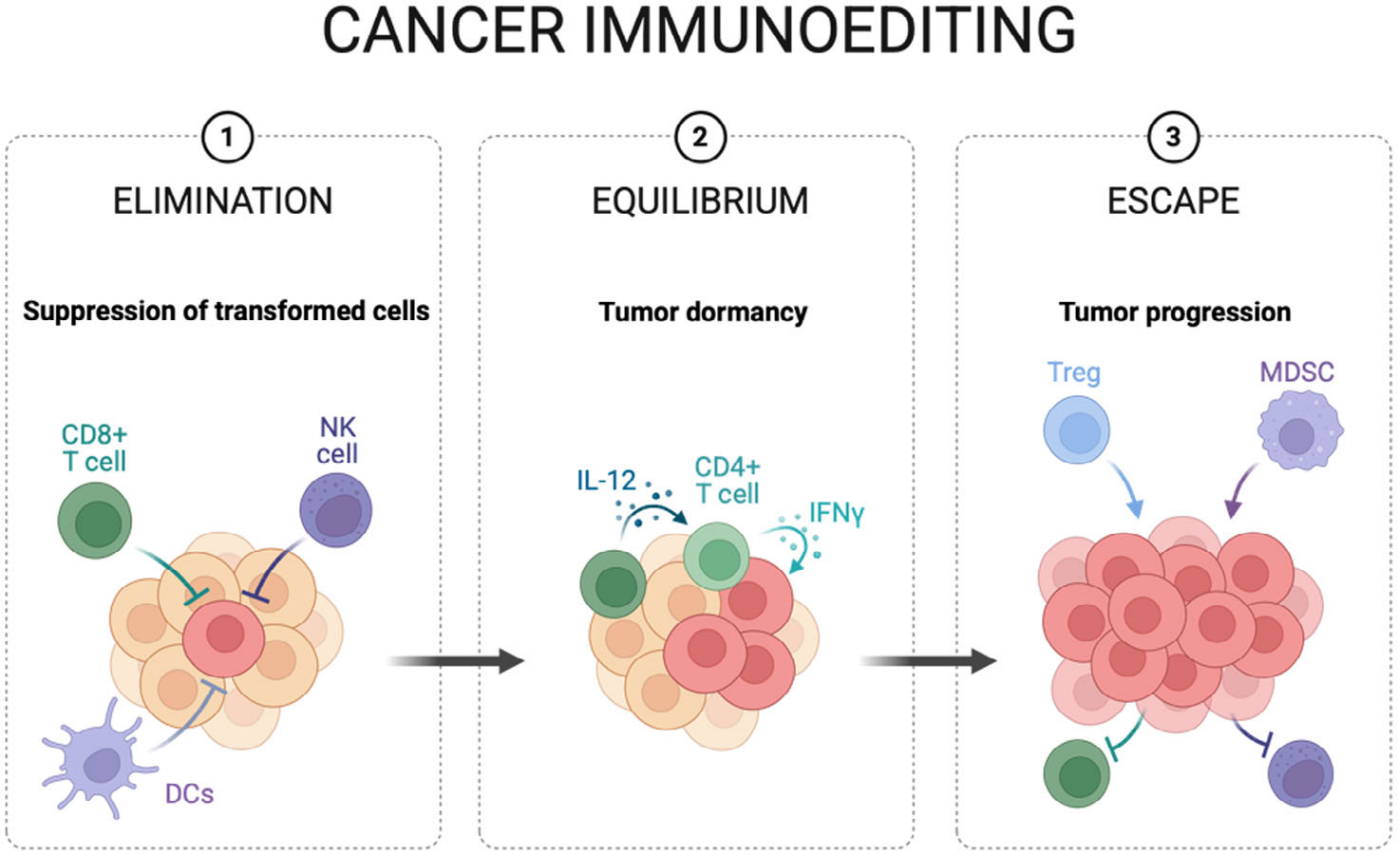
The three phases of cancer immunoediting: 1) Elimination: immune cells target and destroy cancer cells; 2) Equilibrium: the immune system regulates the remaining cancer cells; 3) Escape: cancer cells evade immune surveillance.

Cancer immunoediting (**Figure** **2**), a process that includes the phases of elimination, equilibrium, and escape, illustrates the evolving interaction between the immune system and tumor cells.^[^ ^25^, ^26^ ^]^ In the elimination phase, innate and adaptive immune cells, such as natural killer (NK) cells, dendritic cells (DCs), and cytotoxic T lymphocytes (CTLs), work together to identify and eliminate tumor‐associated antigens (TAAs) and tumor‐specific antigens (TSAs). During the equilibrium phase, immune pressure leads to the selection of tumor variants with diminished immunogenicity, and in the escape phase, tumors develop mechanisms to suppress immune responses. These mechanisms include immune checkpoint modulation (e.g., PD‐1, CTLA‐4), secretion of immunosuppressive cytokines, and alteration of the tumor microenvironment (TME) to encourage immune tolerance.^[^ ^27^ ^]^

Recent findings have broadened our understanding of tumor immunology, highlighting the role of chronic inflammation in cancer progression. Ongoing inflammation, fueled by pro‐inflammatory cytokines, chemokines, and immune cells, creates a microenvironment that supports tumor proliferation, angiogenesis, and metastasis.^[^ ^28^, ^29^ ^]^ Additionally, dysregulation in DNA damage repair pathways, particularly those involving the Mre11 complex, can result in the persistent activation of innate immunity, unintentionally fostering oncogenesis.^[^ ^30^ ^]^ The significance of tertiary lymphoid structures (TLS) and tumor‐infiltrating B cells has also been emphasized in recent studies, showing their potential to influence immune responses and associate with improved clinical outcomes.^[^ ^31^, ^32^ ^]^

Despite significant advancements, immune checkpoint inhibitors (ICIs) targeting PD‐1, PD‐L1, and CTLA‐4 demonstrate limited efficacy in a subset of patients due to both inherent and acquired resistance.^[^ ^33^ ^]^ Tumors frequently adapt by reshaping the tumor microenvironment (TME) to recruit immunosuppressive cells, including regulatory T cells (Tregs) and myeloid‐derived suppressor cells (MDSCs), which suppress antitumor immune responses. Addressing these challenges necessitates innovative strategies to modulate the TME, such as reprogramming tumor‐associated macrophages (TAMs) from a pro‐tumorigenic (M2) to an antitumor (M1) phenotype, targeting metabolic reprogramming in immune cells, or disrupting stromal and vascular components that facilitate immune evasion.^[^ ^34^, ^35^ ^]^ Given these complexities, there is an urgent need for innovative approaches capable of modulating immune responses and overcoming immune suppression in the TME. Inorganic nanomaterials appear to have the potential to fulfill this need due to their immunomodulatory activity, which may enhance antitumor immunity within TME.^[^ ^36^, ^37^ ^]^

Readers who are specifically interested in the interaction between the immune system and cancer may refer to other sources, where these topics have been discussed comprehensively.^[^ ^38^, ^39^, ^40^, ^41^ ^]^

## 3 Noble Metal Nanomaterials: General Features

**Figure 3:**
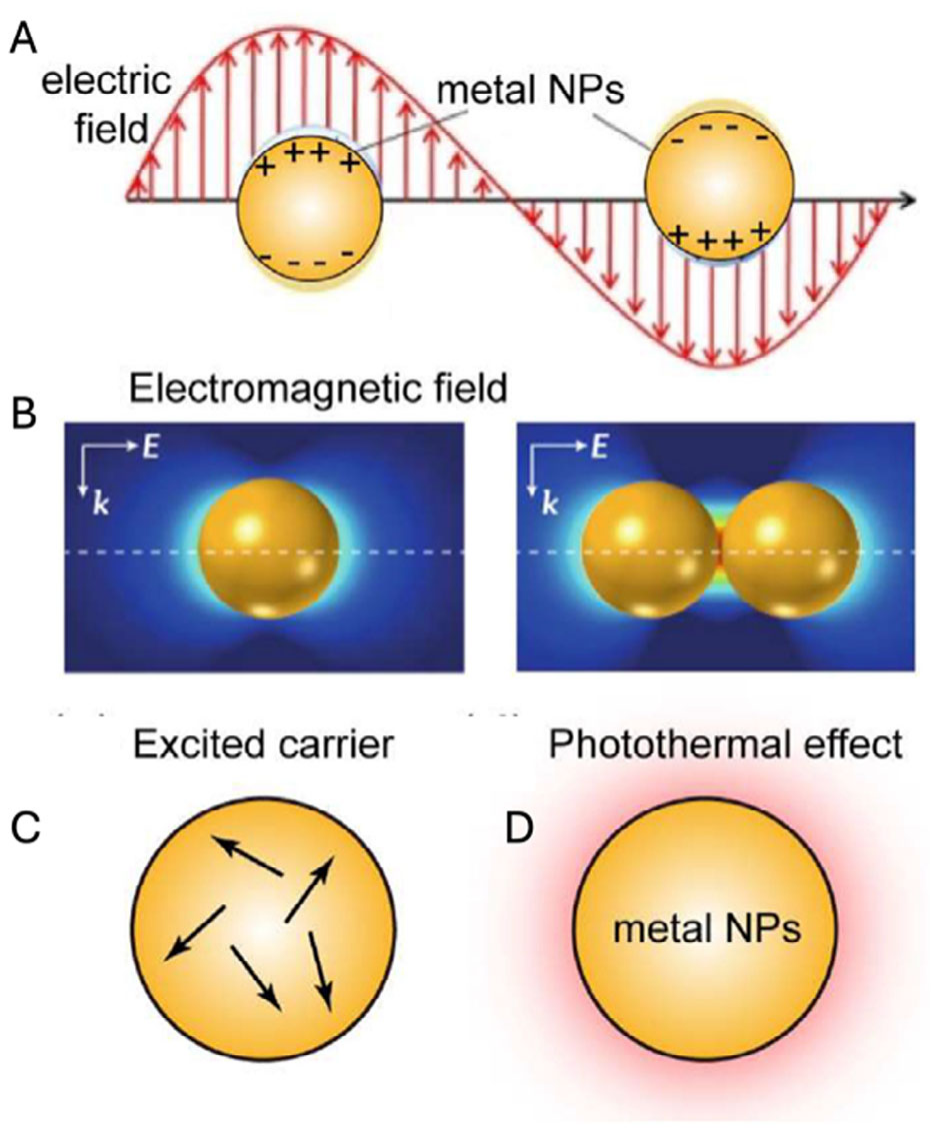
A) Localized surface plasmon resonance (LPSR) of a dipole, B) Electromagnetic field enhancement localized around a single nanoparticle and in coupled nanoparticles with ultrasmall gap distance, C) generation and relaxation of excited carriers upon interaction with light, D) photon energy conversion to thermal energy following excitation. Reproduced with permission.^[^ ^55^ ^]^ Copyright© 2022 Elsevier Ltd. All rights reserved.

Noble metal nanoparticles (NPs) colors are known from ancient times, when gold and silver NPs were unintentionally synthetized during the decoration of glasses.^[^ ^42^ ^]^ Following, Faraday was the first to synthesize noble metal NPs (gold colloids) for scientific purposes while the employment of metal colloids (silver) in clinics is dated back to 1918 to treat a case of puerperal septicemia.^[^ ^43^, ^44^ ^]^ To date, the unique physical, chemical, and physiological features exhibited by noble metal NPs have attracted significant attention, especially for applications in the health sector.^[^ ^7^, ^9^, ^45^ ^]^ Their general features include peculiar light‐matter interactions (such as the localized surface plasmon resonance, LSPR), biocompatibility, and ease of functionalization, which contribute to their versatility in potential applications ranging from imaging to mono‐ and multi‐modal non‐invasive treatments in oncology and beyond.^[^ ^46^, ^47^, ^48^, ^49^ ^]^ The LSPR is particularly pronounced in gold, silver, and copper NPs whereas most other transition metals show only a broad and poorly resolved extinction band in the UV region. It occurs when the frequency of an incident electromagnetic field matches the frequency of an intrinsic electronic oscillation of the metal (**Figure** **3**). The collective oscillation of the electron cloud causes a coherent displacement of the electrons from the nuclei, leading to the formation of distributions of the nanostructure surface charges, such as dipole or quadrupole.^[^ ^50^ ^]^ Each type of surface charge distribution is characterized by a specific resonance energy, i.e., the LSPR. When an incoming radiation of an appropriate frequency interacts with the nanostructure, its energy can be stored in the oscillation mode of the nanoparticle and can result in an absorption and/or in light scattering.^[^ ^50^ ^]^ Thus, the oscillation of the conduction electrons in resonance with the incident light results in unique optical properties.^[^ ^47^, ^50^, ^51^, ^52^ ^]^ LSPR provides the usual vivid colors of noble metal NPs solutions and enhances the electromagnetic fields on the particle's surface, making them highly sensitive to environmental changes and supporting nonlinear optical phenomena.^[^ ^46^, ^48^, ^53^, ^54^ ^]^

This property is critical for bio‐imaging (as in photoacoustic), where the nanoparticles can be tracked using non‐invasive techniques, in biosensing, where even minor molecular interactions can be detected, and in photothermal treatment, where NPs can be used as light‐heat transducers.^[^ ^56^, ^57^, ^58^, ^59^, ^60^ ^]^ Furthermore, due to the high atomic number (**Table** **1**), noble metal NPs intensify the radiation dose deposition within targeted tissues upon exposure to ionizing radiations by acting as radiosensitizers.^[^ ^45^, ^61^, ^62^ ^]^

On top of that, nanotherapeutics based on noble metals can be composed to include more than one action modality in a single platform, i.e., a multimodal therapeutic (such as theranostics, platforms with multiple therapeutic actions, or systems with a guided activity).

The biocompatibility of gold NPs arises from the intrinsic chemical stability, meaning they do not readily dissolve or degrade in a biological environment (Table 1). Indeed, the meaning of “noble metal” for gold is associated with its low reactivity. Due to this feature, gold is approved as a food additive in Europe (additive E175), and it is employed in some food preparation (as in the Vark) or as a component of some alcoholic drinks.^[^ ^63^ ^]^ On the other hand, the special stability of gold is also the cause of one of the prominent issues associated with its clinical translation: the persistence.^[^ ^8^ ^]^ Indeed, the non‐biodegradable nature of gold results in the indefinite residence of NPs in excretory organs, such as the liver. Therapeutics, instead, cannot leave residues in patients after the action as recommended by the approbation agencies. This issue has hindered the translation of gold NPs to the clinical practice in the last decades, but, recently, new breakthroughs have emerged to overcome this drawback, among which the ultrasmall‐in‐nano approach for the design of translational nanotherapeutics.^[^ ^57^, ^61^ ^]^ Silver and copper NPs, instead, can be slowly dissolved in physiological fluids due to the surface oxidation that releases the respective ions.^[^ ^49^, ^64^ ^]^ This process is at the basis of their antimicrobial properties and improves their excretion trends (even if it supports both the interaction with endogenous proteins and the transient redistribution in the organism).^[^ ^11^, ^49^, ^65^ ^]^ In this regard, nanomaterials are readily opsonized in living organisms resulting in the formation of the protein corona on their surface.^[^ ^66^, ^67^, ^68^ ^]^ This phenomenon can be partially modulated by modifying the nanomaterials surface with functional groups, proteins, drugs, or ligands, allowing them to interact specifically with biological targets.^[^ ^69^, ^70^, ^71^ ^]^ The modification of noble metal surfaces is generally straightforward and enables the customization of NPs for specific medical applications such as targeted drug delivery, immunotherapy, or multimodal treatments.^[^ ^72^, ^73^, ^74^, ^75^ ^]^ Besides that, the modification of the surface of noble metal NPs by specific polymers can enhance their accumulation in the target tissue/cells by altering their pharmacokinetics due to the enhanced permeability and retention effect (EPR) observed in some carcinoma.^[^ ^76^, ^77^, ^78^ ^]^ Complementary to the EPR, NPs may be engineered to also modulate the active transport and retention (ATR), i.e., active tissue accumulation mediated by endothelial cells, vesicular‐vacuolar organelles and/or engagement with circulating immune cells followed by NPs trafficking and neoplasm retention mediated by tumor‐associated macrophages (TAMs).^[^ ^79^, ^80^ ^]^ Overall, noble metal NPs have the potential to shift the paradigm in various biomedical applications from diagnostics to targeted therapy and non‐invasive treatments due to the combination of their unique physical, chemical, and physiological properties. This potential is still to be fully validated in human, but it is also increasingly close to the translation thanks to the efforts recently profuse on addressing some key challenges such as: i) investigating their long‐term toxicity, ii) shedding light on their biodistribution, metabolism, and elimination, iii) exploring the interaction with the immune system, and iv) establishing standardized operative procedures, scale up and good manufacturing procedures for their production.

**Table 1:** Physicochemical properties of the bulk metals.

Property	Gold [Au]	Silver [Ag]	Copper [Cu]
Atomic number	79	47	29
Atomic weight	196.97	107.87	63.55
Melting point	1064 °C	961.8 °C	1085 °C
Density	19.32 g cm^−3^	10.49 g cm^−3^	8.96 g cm^−3^
Electrical conductivity	45.2 × 10^6^ S m^−1^	62.1 × 10^6^ S m^−1^	59.6 × 10^6^ S m^−1^
Thermal conductivity	318 W m^−1^K^−1^	429 W m^−1^K^−1^	398 W m^−1^K^−1^
Appearance	Shiny yellow metallic	Shiny white metallic	Shiny reddish‐brown
Chemical reactivity	Very stable, highly resistant to oxidation and corrosion	Moderately reactive, tarnishes with sulfur	Reactive, forms oxides and sulfides in moist air

## 4 Nanomaterials‐Driven Immunomodulation

### 4.1 Gold Nanomaterials

**Figure 4:**
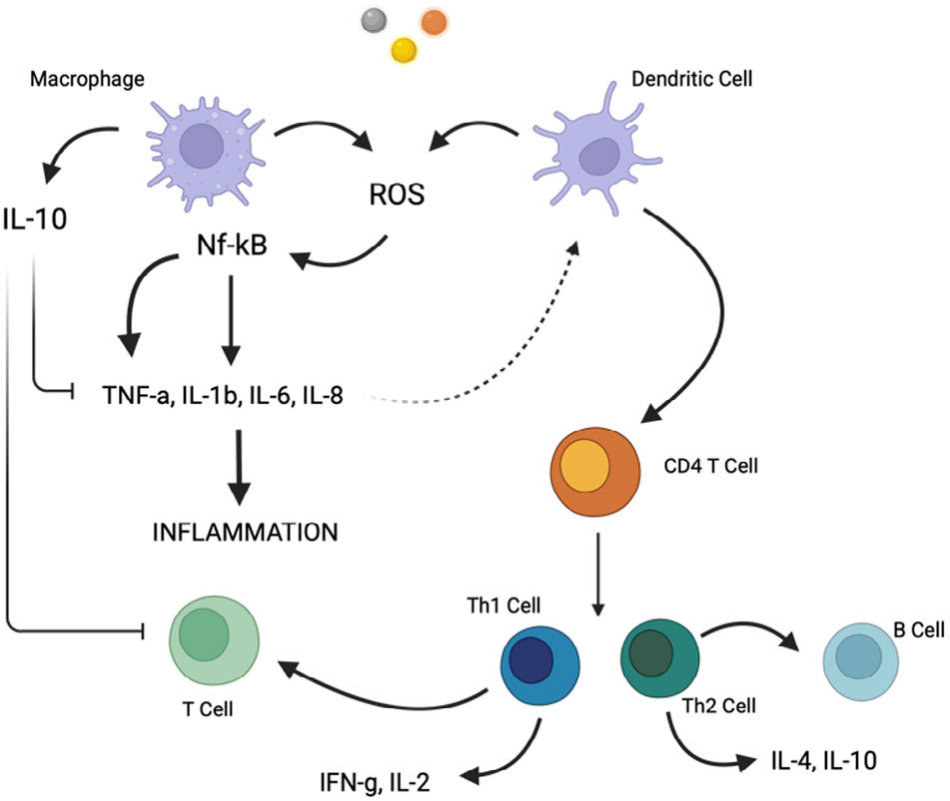
Immune effect mediated by metal‐based nanoparticles on macrophages and dendritic cells.

Gold nanoparticles (AuNPs) stimulate a strong interest in the scientific community because of their peculiar properties, which are advantageous for medical and non‐medical applications.^[^ ^81^ ^]^ AuNPs are generally non‐toxic and biocompatible, addressing the main prerequisites for a therapeutic.^[^ ^82^ ^]^ Nowadays, several synthetic approaches have been identified to obtain a plethora of AuNPs, with different sizes, shapes, and surface decorations.^[^ ^83^, ^84^ ^]^ In the market, AuNPs are employed in sensing applications while they are still not exploited in the therapeutic field, such as clinical oncology.^[^ ^83^, ^84^, ^85^ ^]^ Indeed, their translation to the market requires an accurate evaluation of several safety behaviors, among which the full adsorption‐distribution‐metabolism‐excretion‐toxicity (ADMET) and the nano/immune‐interaction.^[^ ^9^, ^14^, ^86^ ^]^ Usually, macrophages and DCs are the most common lines employed to investigate the effects of metal nanoparticles on the immune system, such as the intracellular uptake and the cytotoxicity (**Figure** **4**).

**Figure 5:**
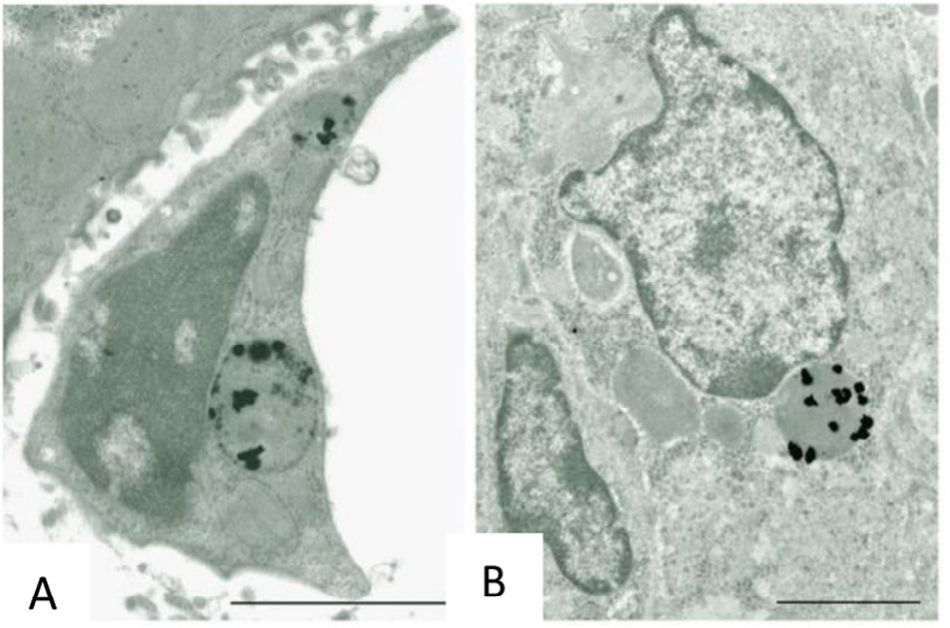
Electron micrographs depict AMG‐enhanced aggregates of AuNPs localized within the lysosomes of a Kupffer cell (A) and a macrophage in the spleen (B). The subject animal was administered intravenously with 40 nm gold nanoparticles and observed for 24 h post‐injection. Scale bars: 2 µm. Reproduced with permission.^[^ ^88^ ^]^ Copyright© 2007, Sabauskadas et al; license BioMed Central Ltd.

In this regard, Zhang et al. evaluated the cellular uptake of AuNPs in cultured RAW264.7 murine macrophage cells.^[^ ^9^ ^]^ Their results showed that macrophages internalize AuNPs into intracellular vacuoles via phagocytosis. Through atomic force microscopy (AFM), confocal fluorescence laser scanning microscopy (CFLSM), and transmission electron microscopy (TEM) analysis, Shukla et al. demonstrated that AuNPs (7–35 Å) can enter cells and then be trapped in vesicles but that are not able to enter the nucleus (**Figure** **5**).^[^ ^87^ ^]^

Pascual García et al. developed and applied a convenient approach based on electron and ion microscopy techniques to analyze the cellular internalization of AuNPs in THP‐1 cells.^[^ ^89^ ^]^ THP‐1 are human leukemia monocytic cells that have been extensively used to explore monocyte/macrophage functions.^[^ ^90^ ^]^ According to this study, AuNPs uptake strongly depends on the size of the NPs. The Authors observed that larger‐size AuNPs (35 nm) can be found dispersed on the cell membrane or aggregated inside the cells, while smaller‐size AuNPs (5 nm) are present as closer aggregates only on the cell surface.^[^ ^89^ ^]^ The importance of size in cellular uptake has been underlined also by Fernandez et al.^[^ ^91^ ^]^ Their studies have shown an enhanced cellular uptake of smaller AuNPs (1–2 nm) by DCs compared to the larger ones (12 nm). An opposite scenario has been identified by Tomic et al., who demonstrated that 50 nm AuNPs were internalized more efficiently by DCs than 10 nm AuNPs.^[^ ^92^ ^]^ These data seem to contradict, but the size‐dependence of AuNPs on the cellular uptake is probably not linear.^[^ ^93^ ^]^

The size plays a fundamental role in AuNPs toxicity because it is associated with the intracellular uptake.^[^ ^94^, ^95^ ^]^ Marche et al. demonstrated that AuNPs cannot induce toxicity in macrophages and DCs after their internalization.^[^ ^18^ ^]^ In their study, both macrophages and DCs were exposed to different concentrations of AuNPs, and no significant cytotoxicity was observed. Through two different assays, Zhang et al. demonstrated that the administration of AuNPs does not affect cell viability or cell membrane integrity in macrophages.^[^ ^96^ ^]^ Simon et al. assessed the toxicity of AuNPs on four different cell lines including macrophages.^[^ ^97^ ^]^ The findings underline a strong dependence on size and a more toxic effect of small AuNPs (1‐2 nm) than the bigger ones (15 nm). A similar scenario has been identified by Hsu et al. In their work, the Authors showed that small differences in the size of NPs produce very different effects.^[^ ^98^ ^]^ As already mentioned, many studies have been carried out using macrophages and DCs as models. In particular, the latter have a key role in the innate and adaptive immune system.^[^ ^18^ ^]^ Both these cells are responsible for the secretion of several cytokines involved in the immune processes. Macrophages are one of the most abundant sources of inflammatory cytokines which are synthesized and released in response to the activation of pattern recognition receptors or inflammasomes.^[^ ^99^ ^]^ DCs produce cytokines, and they are themselves susceptible to cytokine‐mediated activation. They express a unique set of costimulatory molecules that permit the activation of naïve T cells initiating the adaptive response (Figure 4).^[^ ^100^ ^]^ Many research groups focused their attention on the AuNPs ability to increase or reduce the cytokine secretion, which are the main actors in the immune system. In this case, among all the cytokines produced during an immune response, the most investigated are the interleukin (IL)‐1β, IL‐6, Tumor necrosis factor alpha (TNF‐α), and IL‐12p70. Both macrophages and DCs can secrete these important mediators which are responsible for a pro‐inflammatory effect.^[^ ^100^ ^]^ The interaction between AuNPs and macrophages often results in an immunosuppressive effect (**Table** **2**) after the downregulation of the above‐mentioned cytokines. This behavior has been confirmed by Valenzuela et al. According to their investigations, the administration of AuNPs results in an inhibitory effect on macrophages that is confirmed by a reduction in the secretion of TNF‐α and IL‐1β (associated, in turn, to the downregulation of the mRNA expression).^[^ ^101^ ^]^ The immunosuppressive effect is prevalently due to the change in macrophage activity rather than on their number. The work of Huan‐Yao Lei et al. shows a strong inhibition of TNF‐α and IL‐6 production after the interaction between macrophages and AuNPs.^[^ ^102^ ^]^ The researchers demonstrated a correlation between the immunosuppressive effect and the attenuation of unmethylated cytosine‐phosphate‐guanine (CpG) induced Toll‐like receptor 9 (TLR9) functions which are involved in pro‐inflammatory processes. Meyer et al. analyzed the immunosuppressive effect of AuNPs synthesized from *Hypoxis hemerocallidea* derivatives, a wild tuberous plant that is native to the southern parts of Africa. The effect of AuNPs has been evaluated in macrophages and NK cells.^[^ ^103^ ^]^ Both cellular lines have been treated with two different classes of AuNPs: AuNPs synthesized from *H. hemerocallidea* extract and AuNPs synthesized by using hypoxoside, the plant's major secondary metabolite. The interaction between macrophages and the two classes of AuNPs resulted in a significant decrease in IL‐1β and TNF‐α levels. On the other hand, NK cells were sensitive to only AuNPs synthesized from hypoxoside. The effect observed in natural killer cells is a reduced interferon‐gamma (IFN‐γ) production, which represents the major cytokine effector secreted from these cells in response to an infection. It should be noted that in this study, the pre‐activation of the immune cells with lipopolysaccharide (LPS) was performed before the AuNPs administration. Indeed, LPS is a strong activator of the immune system that is usually employed to stimulate an immune response. Pre‐treatment with LPS is a variable that has to be considered to avoid misinterpretation of the findings. Indeed, it is often unclear if the cells are pre‐treated with LPS to investigate the immunomodulatory effect of AuNPs. In this regard, Marche et al. compared the immunostimulatory effect of AuNPs on LPS‐stimulated and unstimulated macrophages and DCs.^[^ ^18^ ^]^ An immune response has been observed only in the cells treated with LPS. AuNPs caused an immunostimulatory effect on macrophages, including an increase in TNF‐α and monocyte chemoattractant protein‐1 (MCP‐1) production. A positive effect was also observed when the DCs were treated with AuNPs. In this case, the immunostimulatory effect includes an increase of IFN‐γ, IL‐13, and IL‐17, reflecting the T cell activation (Figure 4). The interaction between AuNPs and DCs leads to an increased expression of cluster of differentiation 86 (CD86) and major histocompatibility complex II (MHC‐II) which are two markers expressed by antigen‐presenting cells (APCs). Tsai et al. identified a similar macrophage response after AuNPs administration.^[^ ^98^ ^]^ In their study, an up‐regulation of IL‐1β, IL‐6, and TNF‐α gene expression associated with increased cytokine secretion has been observed. Khan et al. evaluated the immune response after AuNPs treatment in liver, spleen, and kidney macrophages. Furthermore, the mRNA expression of specific proinflammatory cytokines has been analyzed in mice exposed to AuNPs.^[^ ^104^ ^]^ In agreement with Tsai et al., they observed an immunostimulant effect in the spleen and liver due to an increase in IL‐1β and IL‐6 expression, while a very small down‐regulation of IL‐1β, IL‐6, and TNF‐α has been identified in the kidney's macrophages.^[^ ^105^ ^]^ The studies carried out on DCs showed, in most cases, an immunostimulatory effect (Table 2). For example, Rong‐Fu Wang et al. showed an increase in IL‐1β, IL‐6, and TNF‐α secretion associated with AuNPs administration.^[^ ^106^ ^]^ On the other hand, Marche et al. demonstrated an opposite immune response with a similar set‐up characterized by the inhibition of the IL‐12p70 secretion that leads to the T lymphocyte activation.^[^ ^107^ ^]^  The interaction between lymphocytes and AuNPs represents another important topic to investigate as they belong to the adaptive immunity cells. Devanabanda et al. focused on the effect of AuNPs on peripheral blood lymphocytes.^[^ ^108^ ^]^ They recognized a significant inhibition of mitogen‐stimulated proliferation of lymphocytes after AuNPs administration. Many investigations focused on the B cell's response after AuNPs treatment. In general, an immunostimulatory effect has been identified. Lee et al. demonstrated that AuNPs can be used to enhance the immunoglobulin G (IgG) secretion by B lymphocytes globally.^[^ ^109^ ^]^ The experiments carried out indicate that AuNPs treatment upregulated B‐lymphocyte‐induced maturation protein 1 (blimp1) – which positively regulates the secretion of IgG – but downregulates paired box 5 (pax5) – responsible for a negative impact on IgG production. A different pathway involved in B lymphocyte regulation has been analyzed by Sharma et al., who observed an increase in IgA production led to the effect of AuNPs on Nuclear Factor κB transcription in B cells (NF‐κB) signaling, which is strongly involved in immune responses.^[^ ^110^ ^]^  Pan et al. demonstrated that minimal size variation between AuNPs produces significantly different cell responses.^[^ ^97^ ^]^ Tsai et al. reported that the expression levels of cytokines from macrophages incubated with AuNPs are considerably affected by the nanoparticles’ diameter.^[^ ^98^ ^]^ The Authors evaluated the immunological response produced in macrophages by AuNPs with three different sizes (small, medium, and large). Briefly, the smallest AuNPs (2–4nm) are associated with a stronger stimulation of the macrophages recognized by the up‐regulation of the gene expression of IL‐1β, IL‐6, and TNF‐α. Khan et al. confirmed these findings by analyzing similar genes.^[^ ^104^, ^105^ ^]^ Lee et al. focused their attention on B lymphocytes and, in agreement with the results on macrophages, observed a size‐dependent enhancement of IgG secretion in response to AuNPs administration.^[^ ^109^ ^]^ By decreasing the size of AuNPs the stimulation increases, and a maximum effect was reached with 2–12 nm AuNPs. Experiments with DCs have been reported by Wang et al., which recognized an immunostimulant effect of AuNPs with a size‐dependent activation of innate signaling pathways.^[^ ^106^ ^]^ In particular, AuNPs <10 nm increased the IL‐1β production by activating the nucleotide‐binding domain, leucine‐rich–containing family, pyrin domain–containing‐3 (NLRP3) inflammasome while AuNPs >10nm lead to an increase of IL‐6 and TNF‐α production through NF‐kB pathway activation.^[^ ^106^ ^]^

**Table 2:** Gold nano/immune‐interaction (pink: immunostimulation, light blue: immunosuppression).

Experiment type	Cell lines/ Animal models	Size	Coating	Effect	Ref.
In Vitro	Murine macrophages (J774.1A)	24.4 ± 0.3 nm (water); 97 ± 7.3 nm (Dulbecco's Modified Eagle Medium (DMEM)) (DLS)	Citrate	↑ TNF‐α and MCP‐1 production	[18]
In Vitro	Murine dendritic cells (C5BL/6)	97 ± 7.3 nm (DLS)	Citrate	Activation of antigen‐specific responses of all T cells: Th1, Th2, Th17 and so respectively ↑ IFN‐γ, IL‐13, and IL‐17 production	[18]
In Vitro	Murine macrophages (J774.1A)	2.8 ± 0.8 nm (Small); 5.5 ± 0.9 nm (Medium); 38.1 ± 11.8 nm (Large) (DLS)	‐	↑ TNF‐α, IL‐1β and IL‐6 production	[98]
In Vitro	Murine macrophages (C57Bl/6)	21.3 ± 0.7 nm (SEM)	Citrate	↓ TNF‐α and IL‐6 expression	[101]
In Vitro	Murine macrophages (RAW264.7)	3.8 ± 0.6 nm, 11.3 ± 1.3 nm, 19.2 ± 2.1 nm, 35.4 ± 5.6 nm, 45.0 ± 4.3 nm (DLS); 4 nm, 11 nm, 19 nm, 35 nm and 45 nm (TEM)	Citrate	TLR9 inhibition and consequently no secretion of IL‐6, IL‐12p70, and TNF‐α	[102]
In Vitro	Human leukemic monocyte cell line (THP1)	26 ± 2.0 nm (DLS)	‐	↓ IL‐1β, IL‐6 and TNF‐α production	[103]
In Vitro	Human natural killer cells (NK92)	26 ± 2.0 nm (DLS)	‐	↓ IFN‐g production	[103]
In Vitro	Murine dendritic cells (C57BL/6)	13, 30, and 70 nm (TEM)	PEG	NF‐kB signaling pathway activation	[106]
In Vitro	Murine dendritic cells (C57BL/6)	4.5 nm (TEM)	PEG	NLRP3 inflammosome activation	[106]
In Vitro	Murine dendritic cells (C57BL/6)	10 nm (TEM)	Citrate	↓ IL‐12p70 secretion	[107]
In Vitro	Murine splenic lymphocytes (C57BL/6J)	50 ± 5.0 nm (Average size, NTA)	Citrate	Inhibition of lymphocytes mitogen‐stimulated proliferation	[108]
In Vitro	Human splenic lymphocytes (Primary cells)	50 ± 5.0 nm (Average size, NTA)	Citrate	Inhibition of lymphocytes mitogen‐stimulated proliferation	[108]
In Vitro	B‐cells isolated from spleen (BALB/C mice)	2, 5, 8, 12, 17, 37, and 50 nm (TEM)	Citrate	Activation of B‐cells and ­ IgG production	[109]
In Vitro	Murine antibody‐generating cells against haptoglobin (5B1B3 B cells, splenocytes fused with myeloma cells)	2, 5, 8, 12, 17, 37, and 50 nm (TEM)	Citrate	↑ IgG production	[109]
In Vitro	Murine B‐lymphocyte (CH12.LX)	9.7 ± 0.1 nm (DLS), 9 ± 1.5 nm (TEM)	Citrate	NF‐kB signaling pathway activation and ­ IgA production	[110]
In Vitro	Human leukemia monocytic macrophages (THP‐1)	5, 15, 20, and 35 nm	Citrate	↓ IL‐1β induced inflammatory response and ↓ TNF‐α production	[111]
In Vitro	Murine macrophages (RAW264.7)	15 nm (TEM)	CpG	TLR9 activation and ↑ IL‐6, IL‐12 and TNF‐α production	[118]
In Vitro	Murine macrophages (RAW264.7)	13 nm (TEM)	CpG	TLR9 and NF‐kB pathways activation	[119]
In Vitro	Murine bone marrow macrophages (BALB/c mice)	20.5 nm (DLS)	AGIP	↑ IL‐1β, IL‐6 and TNF‐α production	[120]
In Vitro	Murine bone marrow macrophages (BALB/c mice)	19.2 nm (DLS)	SAP	↑ IL‐1β, IL‐6 and TNF‐α production	[120]
In Vitro	Murine macrophages (RAW264.7)	10–15 nm (TEM)	PEG	Inhibition of NO production by down‐regulating iNOS transcription levels	[121]
In Vitro	Human leukemia monocytic macrophages (THP‐1)	16.9 ± 2.5 nm, 44.2 ± 6.1 nm (TEM)	‐	↓ IL‐1β and TNF‐α production	[122]
In Vivo	4‐week‐old C57BL/6 male mice	5, 15, 20, and 35 nm	Citrate	↓ IL‐1β induced inflammatory response and ↓ TNF‐α production	[111]
In Vivo	6‐week‐old male BALB/c mice	13 nm (TEM), 27.6 ± 6.6 nm (DLS)	PEG	↑ IL‐1β, IL‐6, IL‐10 and TNF‐α secretion	[116]
In Vivo	6‐week‐old male BALB/c mice	14.8 ± 3.3 nm (DLS), 4 nm (TEM) and 122.6 ± 29.7 nm (DLS),100 nm (TEM)	PEG	Induce acute inflammation	[117]

On the other hand, some works report that AuNPs can exert a size‐dependent immunosuppressive effect with a trend similar to the immunostimulation. Sumbayev et al. evaluated the IL‐1β secretion in macrophages observing that its inhibition was triggered by administering 5 nm AuNPs.^[^ ^111^ ^]^ Tsai et. al recognized a similar effect by analyzing TNF‐α and IL‐6 in macrophages (a more pronounced inhibition of cytokines secretion after the administration of 4 nm AuNPs). Overall, the data reported in the literature indicate that reducing AuNPs size is associated with a more potent (positive and negative) immune response.

Besides size and shape, the coating of AuNPs can affect the nano/immune‐interaction, and some researchers have explored the topic. In general, the coating affects both the cellular uptake and the adsorption of serum proteins onto the AuNPs surface, which results in the formation of the protein corona.^[^ ^101^, ^112^, ^113^ ^]^ Polymers, such as poly(ethylene glycol) (PEG) thiols, are often used as stabilizers for AuNPs to provide biocompatibility and stability.^[^ ^114^, ^115^ ^]^ Cho et al. carried out an investigation by using PEG‐AuNPs to evaluate the inflammatory effect on liver and spleen macrophages in mice.^[^ ^116^ ^]^ Interestingly, an increase of IL‐1β, IL‐6, IL‐10, IL‐12β, and TNF‐α was observed at a non‐toxic concentration of AuNPs. In a subsequent work, the same authors compared the immunomodulation associated to PEG‐AuNPs with two different sizes (4 and 100 nm), noticing that the increase in IL‐1β and IL‐6 secretion is associated to the up‐regulation of the involved genes without significant difference between the two sizes.^[^ ^117^ ^]^ Chen et al. and Odom et al. investigated the immunostimulatory activity of cytosine‐phosphate‐guanine (CpG)‐conjugated AuNPs.^[^ ^118^ ^]^ The unmethylated CpG is a DNA motif naturally present in microbes that can be recognized by the toll‐like receptor 9 (TLR9), an important receptor involved in the immune response.^[^ ^119^ ^]^ Thus, the conjugation of nanomaterials with CpG can be advantageous in improving the stimulation of the immune system. Chen et al. confirmed this theory, recognizing an immunostimulatory effect associated with the increased secretion of IL‐6, IL‐12, and TNF‐α after CpG‐AuNPs administration.^[^ ^118^ ^]^ With the employment of similar nanoparticles, Odom et al. recognized an activation of the NF‐kB signaling pathways that led to the production of several pro‐inflammatory cytokines and observed that small CpG‐AuNPs (13nm) have an increased effect (probably due to an improved targeting ability).^[^ ^119^ ^]^ Bastús et al. focused on AuNPs decorated with peptides and analyzed their effects on cytokine production.^[^ ^120^ ^]^ Two different peptides have been evaluated: amyloid growth inhibitor peptide (AGIP, composed of six amino acids) and sweet arrow peptide (SAP, consisting of 19 amino acids). Both nanostructures determined an increase in the secretion of IL‐1β, IL‐6, and TNF‐α. Interestingly, despite AGIP and SAP are chemically and structurally different, they resulted in similar effects when conjugated to AuNPs, while the building blocks alone (peptides and AuNPs) did not exert any effect. This behavior seems to be associated with the hydrophobic domains that support the peptide packing on the AuNPs surface. On the other hand, the leveling effect caused by the protein corona formation from proteins released in the medium by cells was not considered. Overall, the works analyzed underline the importance of controlling some key characteristics of AuNPs, among which are the size and the surface, to modulate the cellular internalization and, in turn, the effect on the immune system.

It should be noted that, in several works, the AuNPs effect has been evaluated both in vitro and in vivo. For example, Lee et al. employed 5B1B3 mouse hybridoma cells to perform in vitro experiments that have been followed by an investigation with four‐week‐old male BALB/C mice.^[^ ^109^ ^]^ They observed in both biomodels that the AuNPs activate B‐cells and enhance IgG secretion. On the contrary, Sumbayev et al. performed in vitro and in vivo tests observing an immunosuppressive effect of AuNPs on macrophages.^[^ ^111^ ^]^ In this case, THP‐1 human leukemia monocytic macrophages have been used for in vitro tests while the in vivo experiments have been performed on 4‐week‐old C57BL/6 male mice. In both in vitro and in vivo studies, they observed a downregulation of IL‐1β‐induced inflammatory response and reduced production of TNF‐α. In the work of Devanabanda et al., the AuNPs immunomodulatory effect was investigated in vitro using murine splenic and human peripheral blood lymphocytes, and an immunosuppressive impact was observed in both cell lines.^[^ ^108^ ^]^ Elbagory et al. conducted in vitro studies focused their attention on two different cell lines: THP‐1 human leukemia monocytic macrophages and human NK92.^[^ ^103^ ^]^ The results show a reduced production of specific pro‐inflammatory cytokines in both macrophages and natural killers.

### 4.2 Silver Nanomaterials

**Figure 6:**
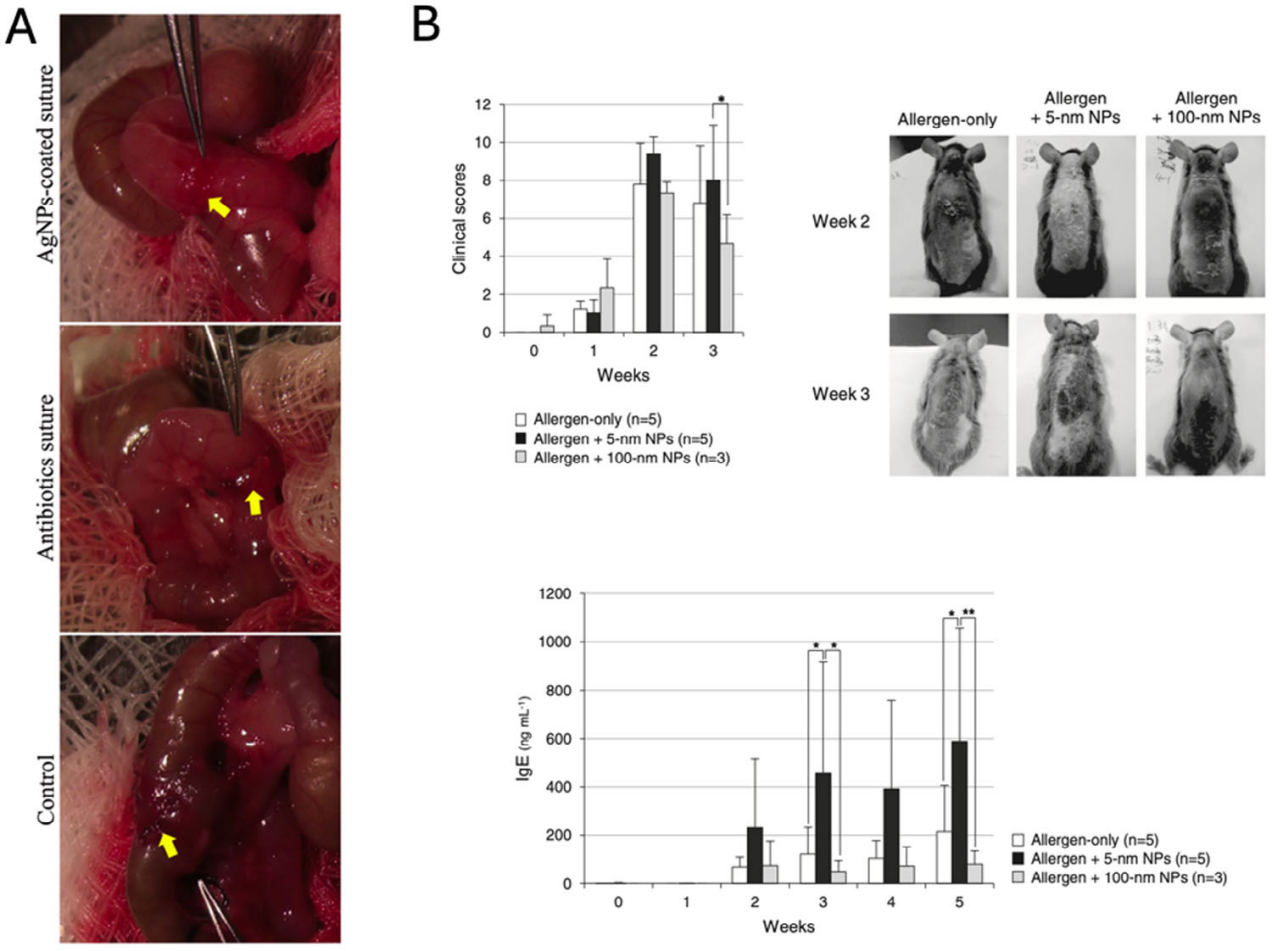
A) Photographs illustrating the macroscopic appearance of intestinal anastomoses (indicated by arrows) in the AgNP‐coated suture group, the antibiotic‐coated suture group, and the control group, on postoperative day 28. Reproduced with permission.^[^ ^146^ ^]^ Copyright© 2017 Elsevier Inc. All rights reserved. B) Evaluation of the clinical skin scores and serum IgE levels in mice with atopic dermatitis. The clinical score (upper‐left) was calculated as the sum of individual scores assigned to four signs and symptoms (erythema/hemorrhage, scaling/dryness, edema, and excoriation/erosion), each graded on a scale of 0 (none), 1 (mild), 2 (moderate), and 3 (severe). Representative images (upper‐right) of skin features from the second and third week are presented. Serum IgE levels (bottom) were measured using a mouse IgE‐specific ELISA kit, with samples diluted to 1:100 and 1:1000. Results are presented as mean ± SD, and statistical analysis was conducted using two‐way ANOVA. *p < 0.05, **p < 0.005. Reproduced with permission.^[^ ^145^ ^]^ Copyright© 2016 Wiley‐VCH Verlag GmbH & Co. KGaA, Weinheim.

Silver nanoparticles (AgNPs) have attracted much interest in biomedical applications due to their antimicrobial activity and potential employment in oncology.^[^ ^74^, ^123^, ^124^, ^125^ ^]^ Besides the biomedical field, the unique physical and chemical properties of AgNPs, among which are low persistence, favorable biodistribution, and biocompatibility, supported their application in the food, cosmetic, and healthcare sectors.^[^ ^126^ ^]^ For example, AgNPs can be found in commercial respirators, household water filters, catheters, cardiovascular implants, food packages, cosmetics, and textiles.^[^ ^11^ ^]^ Several synthetic approaches, including green strategies, have been reported in the literature to obtain stable AgNPs.^[^ ^126^, ^127^ ^]^ Recently, the synthesis mediated by plant extract or microorganisms for producing AgNPs represents two promising approaches for their environmentally friendly production.^[^ ^128^, ^129^, ^130^ ^]^ Once in the bloodstream, AgNPs can interact (**Table** **3**) with lymphocytes (B cells, T cells, and NK) and granulocytes (basophils, eosinophils, neutrophils, mast cells, DCs, and macrophages).^[^ ^131^ ^]^ This interaction results in a size‐dependent immune modulation.^[^ ^132^ ^]^ For example, Park et al. investigated the effect of AgNPs on human macrophages (U‐937) with three different sizes (4, 20, and 70 nm). The AgNPs–macrophage interaction resulted in an immunostimulatory response characterized by an increased expression of IL‐8 (a proinflammatory marker) with the most important effect recorded for 4 nm AgNPs.^[^ ^133^ ^]^ A similar trend has been observed by Lim et al. Indeed, they reported an immunostimulant effect of 5 nm AgNPs on U‐937, while 100 nm AgNPs did not induce any significant effect.^[^ ^134^ ^]^ Also, Nishanth et al. recognized a size‐dependent immune response of macrophages (RAW 264.7) treated with 15 or 40 nm AgNPs.^[^ ^135^ ^]^ The immune response was characterized by increased TNF‐α, IL‐6, and (NF‐kB) expression. Beyond the size dependence, the authors observed a time‐dependent influence on the immune response for both the AgNPs (15 and 40 nm). Briefly, by increasing the time of exposure of the cells to the NPs, the released pro‐inflammatory markers increased. The relationship between size and effect has also been investigated by Yang et al., who carried out a study on primary human monocytes to evaluate the immunological impact of AgNPs on the induction of innate immunity.^[^ ^136^ ^]^ The results showed an immunostimulant response for the AgNPs with sizes of 5 and 28 nm, while NPs of 100 nm did not induce any significant effect. The immunostimulant response included an increase of IL‐1β secretion, and the formation of the NLRP3 inflammasome followed by the activation of the caspase‐1. Alsaleh et al. investigated the immunostimulation of mast cells resulting from the interaction with AgNPs.^[^ ^137^ ^]^ Mast cells are important effectors that regulate both the innate and adaptive immunity.^[^ ^137^ ^]^ The authors performed the experiments using bone marrow‐derived mast cells (BMMC) isolated from C57Bl/6 mice and RBL‐2H3 cells (rat basophilic leukemia cell line). The administration of 20 nm AgNPs resulted in the degranulation of mast cells and, consequently, an increased inflammatory process. On the contrary to these studies, by comparing three different AgNPs sizes (15, 30, and 55 nm) Carlson et al. recorded the most significant response for the NPs showing 55 nm in size. In their study, 55 nm AgNPs increased IL‐1β, TNF‐α, and MIP‐2 secretion in alveolar macrophages.^[^ ^138^ ^]^ Wong et al. evaluated the anti‐inflammatory effect produced by 10 nm AgNPs on two mouse macrophage cell lines (RAW264.7 and J774.1).^[^ ^139^ ^]^ According to this study, the interaction between macrophages and AgNPs reduced TNF‐ α and IFN‐γ secretion. In another study on AgNPs associated with dendrimers (Ag‐DNC), Wong et al. identified a similar immune response in the same macrophage cell lines by analyzing the TNF‐α and IL‐6 reduction.^[^ ^140^ ^]^ These results were further validated in an in vivo study on a burn wound mice model. Moldovan et al. also reported an anti‐inflammatory effect of AgNPs.^[^ ^141^ ^]^ The immune‐suppressive response was identified using a HaCaT cell line and was confirmed by an in vivo study performed on an acute inflammation model of Wistar rats. The immune response was evaluated by recording the decrease of several pro‐inflammatory cytokines (IL‐1α, IL‐6, IL‐10, and TNF‐α). Data collected on cultured cells should always be validated in models of increased complexity, able to mimic the correlation between the physiological phenomena.^[^ ^142^, ^143^, ^144^ ^]^ An example in this direction is the work of Huang et al. They investigated the effects of AgNPs on polymorphonuclear neutrophils (PMNs).^[^ ^145^ ^]^ The in vitro investigations demonstrated an antibacterial activity of AgNPs associated with the activation of neutrophils. On the contrary, the in vivo tests on infected air pouch models resulted in an immunosuppressive effect of AgNPs in which the antibacterial activity of neutrophils was inhibited. In addition, after the in vivo experiment, the inflammatory state of the infected air pouch was explored through an *ex‐vivo* test measuring the level of certain inflammatory markers on the extracted lavage fluid, and a decreased TNF‐α and IL‐6 concentration was observed (**Figure** **6**).

The surface characteristics of AgNPs can strongly modify their biological and physiological features, and subsequently the overall bio/nano‐interactions such as the immunomodulatory effects and the toxicity.^[^ ^136^, ^147^ ^]^ For example, Nguyen et al. compared the impact of naked and coated AgNPs.^[^ ^148^ ^]^ The coatings included in the investigation were citrate and polyvinylpyrrolidone (PVP), and the size of AgNPs considered: 10, 50, and 75 nm. The study generally reported a dependence between the size and the immune modulatory effect. Moreover, the naked AgNPs decreased the expression of selected cytokines including TNF‐α, IL‐1β, and IL‐12 (p70) in J774A cells while AgNPs coated with both citrate and PVP produced an increase of these pro‐inflammatory markers. Between the citrate‐ and the PVP‐AgNPs, the latter induces a more prominent cytokine level increase. This investigation also reported that uncoated AgNPs seem more toxic than coated ones as they increase reactive oxygen species (ROS) production in addition to the immunosuppressive response. On the other hand, in the work of Yilma et al., the PVP is associated with an inhibition of macrophages’ activity.^[^ ^149^ ^]^ All PVP‐AgNPs of 10, 20, and 80 nm down‐regulate a plethora of pro‐inflammatory cytokines and chemokines (TNF‐α, IL‐6, IL‐12 p70, granulocyte‐macrophage colony‐stimulating factor (GM‐CSF), IL‐1α, granulocyte colony‐stimulating factor (G‐CSF), C‐X‐C motif chemokine ligand 10 (CXCL10), and chemokine (C‐C motif) ligand 5 (CCL5)) and the more substantial immunosuppressive effect is associated to the smallest nanostructure.^[^ ^148^ ^]^ Bastos et al. compared citrate‐ and PEG‐coated 30 nm AgNPs. For both AgNPs, an immunosuppressive impact based on the decrease of the MCP‐1 production has been recorded on HaCat cells.^[^ ^150^ ^]^ In another work from Bastos et al., a similar immunosuppressive effect was recognized on 10 nm AgNPs coated by PEG or citrate.^[^ ^151^ ^]^ On the contrary, Balaram Das et al. reported an immunostimulatory effect associated with PEG‐coated AgNPs.^[^ ^152^ ^]^ In addition, the authors investigated bovine serum albumin (BSA) coated AgNPs, and for both the nanoparticles recognized an up‐regulation of pro‐inflammatory mediators (TNF‐α and IL‐12) and a down‐regulation of anti‐inflammatory cytokines (TGF‐β and IL‐10). By comparing the effects, BSA‐AgNPs exhibited a higher activity than PEG‐AgNPs. Tao et al. observed that CpG decorated AgNPs (CpG‐AgNPs) activate macrophages and determine an increase in TNF‐α and IL‐6 secretion. According to this work, the produced immune response is based on the interaction between the coating and the TLR‐9, as confirmed by the absence of immunostimulation from uncoated AgNPs.^[^ ^153^ ^]^

**Table 3:** Silver nano/immune‐interaction (pink: immunostimulation, light blue: immunosuppression).

Experiment type	Cell lines/ Animal models	Size	Coating	Effect	Ref.
In Vitro	Human macrophages (U937)	4, 20, and 70 nm (TEM)	PVP	↑ IL‐8 production	[133]
In Vitro	Human macrophages (U937)	3.7 nm (DLS) and 7.9 ± 5.3 nm (TEM); 96.7 nm (DLS) and 70.9 ± 71.3 nm (TEM)	PVP	↑ IL‐8 production	[134]
In Vitro	Murine macrophages (RAW264.7)	15 ± 3 nm and 40 ± 4 nm (DLS)	Citrate	↑ Cyclooxygenase‐2 (Cox‐2) expression, ↑ TNF‐α, IL‐6 production and induction of NF‐kB translocation	[135]
In Vitro	Human monocytes (Primary cells)	7.9 ± 5.3 nm, 18.3 ± 10.3nm and 81 ± 42.1 nm (DLS)	PVP	↑ IL‐1β production	[136]
In Vitro	Human peripheral blood mononuclear cells (Primary cells)	7.9 ± 5.3 nm, 18.3 ± 10.3 nm and 81 ± 42.1 nm (DLS)	PVP	↑ IL‐ 1β production	[136]
In Vitro	Murine bone marrow‐derived mast cells (C57BI/6)	29.7 ± 0.3 nm (DLS)	Citrate	Mast cells activation	[137]
In Vitro	Murine alveolar macrophages (NR8383 CRL‐2192)	15, 30, and 55 nm (SEM)	Hydrocarbon	↑ TNF‐α, IL‐1β and MIP‐2 production	[138]
In Vitro	Murine macrophages (RAW264.7)	9.3 nm (Average size) (TEM)	Citrate	↓ TNF‐α and IFN‐γ production	[139]
In Vitro	Murine macrophages (J774)	10, 20, and 80 nm	PVP	↓ TNF‐α and IL‐6 production	[149]
In Vitro	IL‐2‐dependent T lymphoblastoid cell line (WE17/10)	10, 20, and 80 nm	PVP	Inhibition cell proliferation mediated by IL‐2	[149]
In Vitro	HaCat cells	25 nm (Average size) (TEM)	Bioactive compounds	↓ IL‐1α and IL‐6 production	[141]
In Vitro	Human PMNs (Primary cells)	17.9 ± 7.8 nm (TEM)	PVP	↓ ROS, IL‐1β and MCP‐1 production and ↓ number of neutrophils recruited to the inflammatory site	[145]
In Vitro	HaCat cells	27.1 ± 3.0 nm (STEM)	Citrate 30	↓ MCP‐1 production	[150]
In Vitro	HaCat cells	27.7 ± 3.2 nm (STEM)	PEG 30	↓ MCP‐1 production	[150]
In Vitro	Human PBMCs (Primary cells)	200 ± 25.0 nm (DLS), 50 ± 10.0 nm (TEM)	PEG	↑ TNF‐α, IL‐12 and ↓ TGF‐β and IL‐10 production	[152]
In Vitro	Human PBMCs (Primary cells)	200 ± 25.0 (DLS), 40 ± 10.0 nm (TEM)	BSA	↑ TNF‐α, IL‐12 and ↓ TGF‐β and IL‐10 production	[152]
In Vitro	Murine macrophages (RAW264.7)	1.5 nm (Average size) (TEM)	CpG	↑ TNF‐α and IL‐6 production	[153]
In Vitro	Human PMNs (Primary cells)	19.9 nm (TEM)	Citrate	Induction of apoptosis and inhibition of *de novo* protein production	[154]
In Vitro	HaCat cells	20‐80 nm (TEM)	Natural polyphenols	↓ IL‐1α, IL‐1β and IL‐6 production	[155]
In Vitro	Murine macrophages (RAW264.7 and J774)	10 nm (Average size) (TEM)	Dendrimers	↓ TNF‐α and IL‐6 production	[140]
In Vitro	Murine macrophages (J774)	27.8 ± 5.2 nm, 52.6 ± 2.4 nm, 71.6 ± 9.8 nm, 89.1 ± 7.9 nm (TEM)	‐	↓ TNF‐α, IL‐1β and IL‐12p70 production	[148]
In Vitro	Murine macrophages (J774)	9.9 ± 1.2 nm, 50.6 ± 4.9 nm, 78.6 ± 8.7 nm (TEM)	Citrate	↑ TNF‐α, IL‐1β and IL‐12p70 production	[148]
In Vitro	Murine macrophages (J774)	11.9 ± 1.5 nm, 52.6 ± 3.2 nm, 78.4 ± 3.0 nm (TEM)	PVP	↑ TNF‐α, IL‐1β and IL‐12p70 production	[148]
In Vitro	Immortalized embryonic mouse microglia (N9 cell line)	49.7 ± 10.5 nm (TEM)	Citrate	↓ Microglial inflammation markers (ROS, nitrite and TNF‐α)	[156]
In Vitro	HCT 15 cell	23.5–60.8 nm (TEM)	‐	↓ Nitrogen monoxide (NO) synthesis	[157]
In Vitro	THP‐1 derived macrophages	10 and 75 nm (TEM)	Citrate	↓ TNF‐α, IL‐1β, IL‐6 and IL‐8	[158]
In Vitro	Human natural killers (Primary cells)	20.6 nm	Citrate	↓ The killing potential of NK cells	[159]
In Vitro	Murine macrophages (RAW264.7)	2.0, 3.4, 5.7, 15.4, and 34.7 nm (Average Size) (DLS)	Hydroxylated polyester dendrimers	Activation TNF‐α promoter	[160]
In Vitro	Human promyelocytic cell line (HL‐60), Human monocytic cells (U‐937)	17 ± 5 nm (TEM)	Poly(allylamine hydrochloride) (PAH)	↑ NO production	[161]
In Vitro	Murine mast cells (RBL‐2H3)	5.1 and 74.2 nm (Average size) (DLS)	PVP	Activation of mast cells and granule release, ↑ IgE production	[162]
In Vitro	Peripheral blood mononuclear cells (THP‐1, NCI‐H460 and HL‐60)	20.2 ± 0.5 nm (water), 22.2 ± 0.6 nm (DMEM) (DLS) 22.0 ± 0.4 nm (TEM)	Polyoxyethylene glycerol trioleate and Polyoxyethylene (20) sorbitan mono‐laurate (Tween 20)	Activation of the complement system, ↑ TNF‐α, IL‐1β, IL‐6, IL‐10, IFN‐γ and GM‐CSF production	[163]
In Vitro	Murine macrophages (J774A.1)	14.2 ± 2.0 nm (TEM)	‐	↑ TNF‐α, IL‐1β, IL‐4, IL‐12p70, Eotaxin and KC production	[164]
In Vitro	ACH‐2 cells	45 nm (Average size) (DLS)	Curcumin	↓ TNF‐α, IL‐1β, IL‐6, and NF‐kB expression	[165]
In Vivo	Hind paw edema in rats	25 nm (Average size) (TEM)	‐	↓ IL‐1α, IL‐6, IL‐10 and TNF‐α production	[141]
In Vivo	Air pouch model (2‐month‐old female Sprague‐Dawley (SD) rats)	17.9 ± 7.8 nm (TEM)	PVP	↓ ROS, IL‐1β and MCP‐1 production and ↓ number of neutrophils recruited to the inflammatory site	[145]
In Vivo	Carrageenan‐induced paw edema in rats	20–80 nm (TEM)	Natural polyphenols	↓ IL‐1a, IL‐1β and IL‐6 production	[155]
In Vivo	C57BL/6N mice	<100 nm	Poly‐ methacrylic acid (PMA)	↓ TNF‐α, IL‐6, and IL‐10 and ↓ amount of macrophages infiltrated	[146]
In Vivo	C57BL/6N mice	18 nm (Average size) (TEM)	PVP	↓ number of DCs	[166]
In Vivo	*Labeo rohita* fish	30 nm (Average size) (TEM)	PVP	↑ IL‐8 production and ↑ Cox‐2 expression	[167]

### 4.3 Copper Nanomaterials

Copper nanoparticles (CuNPs) have gained increasing interest in the biomedical community in the last few years.^[^ ^49^, ^65^, ^168^ ^]^ Indeed, CuNPs have demonstrated promising antibacterial, antifungal, and antiviral activity against a broad spectrum of pathogens, wound healing features, and an impact on metastasis regulation.^[^ ^49^, ^65^, ^169^, ^170^ ^]^ Moreover, compared to other metals such as silver and gold, CuNPs can be biodegraded and dissolved in physiological fluids, supporting a fast and easy metabolization and excretion.^[^ ^49^ ^]^ Overall, by considering the influence CuNPs may have in clinical applications, an increasing effort has been afforded toward the elucidation of CuNPs toxicological profile, even if the information are actually quite scarce and field‐specific.^[^ ^49^, ^65^, ^171^, ^172^, ^173^, ^174^ ^]^ For example, many evaluations regarding the CuNPs effect on the immune response have been performed on marine and farm species, which may accumulate a similar nanoparticulate through the environment (**Table** **4**). Indeed, copper is widely employed in agriculture as a biocides, which explains the potential presence of CuNPs in the environment, including marine ones.^[^ ^175^, ^176^ ^]^ Moreover, copper‐based nanoagrochemicals are increasingly employed as effective nano‐fertilizers to enhance the nutritional status of crops and mitigate the incidence of diseases.^[^ ^177^, ^178^ ^]^ Wang et al. evaluated the effect of CuNPs (10–30 nm) on pupperfish (Takifugu fasciatus) regarding the oxidative stress regulation, apoptosis, and immune response.^[^ ^179^ ^]^ The exploration was carried out on the fish's liver, where an increase in the oxidative stress markers and the apoptosis index was observed. In addition to these effects, some physiological indicators of the immune response such as the heat shock protein 70 (HSP70), heat shock protein 90 (HSP90), immunoglobulin M (IgM), and lysozyme (LZM) resulted in increased levels after the CuNPs exposure. Dawood et al. also recognized an immunostimulatory effect while evaluating the influence of CuNPs on the growth, immunity, and oxidative stress of common carp.^[^ ^180^ ^]^ The researchers tested several concentrations of CuNPs (0, 0.5, 1, 2, and 4 mg CuNPs/kg diets) and the ones higher than 1 mg level resulted in an increase of lysozyme, phagocytic activity, and IgM production. It is interesting to notice that an opposite effect has been described by Delaveri et al. in a study performed on rainbow trout (*Oncorhynchus mykiss*).^[^ ^181^ ^]^ By the measurement of the intestinal gene expression by fluorescent real‐time PCR, they demonstrated a decrease of the TNF‐α, IL‐1β, IL‐10, CAD, SOD, and GPx gene expression. A similar suppressive effect has been observed by Scott et al. with *in‐ovo* experiments carried out using broiler eggs from commercial breeder Ross 308 chickens (37 weeks old).^[^ ^182^ ^]^ This biomodel's choice correlates to the authors' main purpose to evaluate the effect of CuNPs (2–15 nm) and copper sulfate (CuSO_4_) during embryogenesis development. In addition to this study, the authors analyzed the immune response after the exposure to CuNPs, which has been characterized by decreased TNF‐α, NF‐kB, and VEGF mRNA expression. An immunosuppressive effect has also been reported by Devanabanda et al. and Lee et al., who performed, respectively, experiments by using murine lymphocytes and murine splenic mononuclear cells.^[^ ^183^, ^184^ ^]^ In the latter, the inhibition of mitogen‐stimulated spleen‐derived lymphocyte proliferation by CuNPs (32‐43nm) has been observed together with the suppression of B‐ and T‐lymphocyte‐mediated immune responses. The immune response directly depends on the dose administered to the animal models (100, 200, and 400 mg kg^−1^ day^−1^).^[^ ^183^ ^]^ In the work of Devanabanda, the authors report that the administration of CuNPs (23–33nm) determines the suppression of lymphocyte proliferation in the spleen and thymus.^[^ ^184^ ^]^ Various doses have been examinated (1, 2.5, 5, 10 µg mL^−1^) and the higher immune responses has been observed for the higher dose. An opposite scenario has been reported by El‐kazaz et al.^[^ ^185^ ^]^ They analyzed the association between the presence of CuNPs in the drinking water of broilers and their immune status. CuNPs (100 nm) have been administered as a solution (10 mg mL^−1^) and, from blood sample evaluations (at day 35), an increase of IgA, IgG, IgM, and IL‐6 secretion has been recognized. Thus, a general improvement in the broiler's immunity has been observed. A similar response on the same model has been also observed by Wang et al. by introducing CuNPs (85–105 nm) into the daily diet of the broilers at different doses (50, 100, 150 mg kg^−1^).^[^ ^186^ ^]^ It should be highlighted that the CuNPs employed in this investigation were modified on the surface with chitosan, a compound that presents both immuno‐enhancing effects and antibacterial activities. The increase of IgA, IgG, and IgM levels has confirmed the immunostimulation after the treatment with copper‐loaded chitosan nanoparticles (CNP‐Cu). An immunostimulatory effect has also been recognized by Ognik et al. by employing 5 nm CuNPs on chicken.^[^ ^187^ ^]^ The effect was observed from the analysis of blood samples, which confirmed an increase in IL‐6, IgA, IgM, and IgY production, dependent on the copper concentration (5, 10, 15 mg L^−1^).

**Figure 7:**
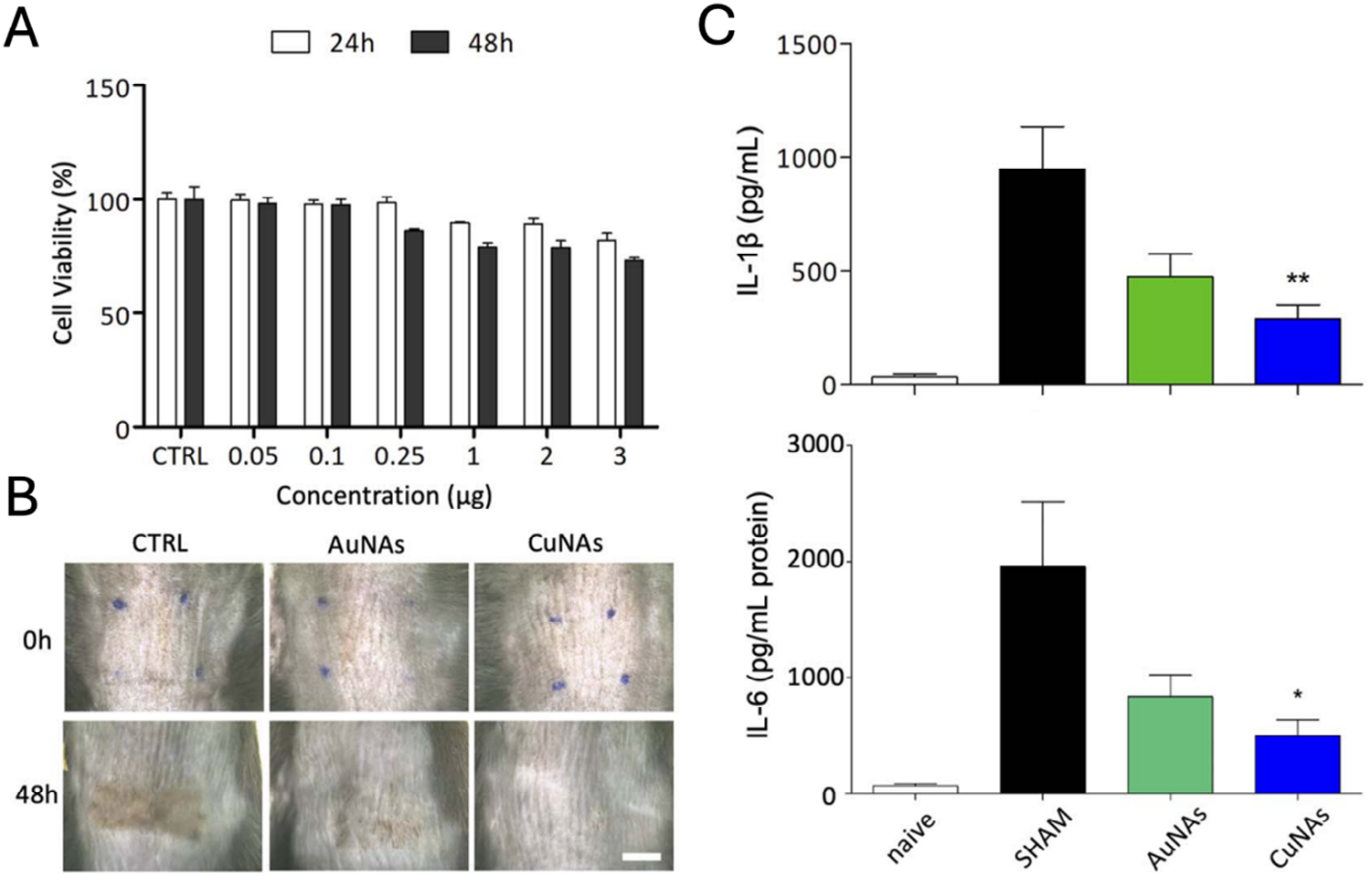
Evaluation of in vitro biocompatibility and efficacy of CuNAs for the treatment of burnt skin. A) The viability of human keratinocyte cells (HaCaT) was assessed after 24 and 48 h of exposure to different CuNAs concentration (reported in µg of copper). Values were normalized to the untreated control group, and results are expressed as mean percentage values ± S.D. from three independent experiments. B) Representative images of UVB‐exposed mouse skin, showing untreated (CTRL) and treated groups with AuNAs or CuNAs. Scale bar: 5 mm. C) The impact of UVB‐induced inflammation on cytokine expression (IL‐6 and IL‐1β) was measured 48 h after the treatment. Results are expressed as mean ± SD (*n* = 4 per group). Statistical analysis was performed using one‐way ANOVA with Bonferroni's post hoc test, where *p < 0.05 and **p < 0.01. Naïve: non‐irradiated, untreated controls; SHAM: irradiated, untreated controls. Reproduced with permission^[^ ^65^ ^]^ Copyright© 2023 The Author(s). Published by the Royal Society of Chemistry.

Finally, a significant anti‐inflammation effect (reduction of the levels of IL‐6 and IL‐1β) has been observed on UVB skin burnt murine models treated with copper‐loaded nano‐architectures (**Figure** **7**).^[^ ^65^ ^]^ In this peculiar nanomaterial, ultrasmall copper nanoparticles of ≈1 nm are loaded in biodegradable silica nanocapsules, and the effect has been recorded after 48h from the treatment.

**Table 4:** Copper nano/immune‐interaction (pink: immunostimulation, light blue: immunosuppression).

Experiment type	Cell lines/ Animal models	Size	Coating	Effect	Ref.
In Vitro	Human splenic mononuclear cells (Primary cells)	2.7 ± 10.4 nm (TEM)	‐	Inhibition of mitogen‐stimulated spleen‐derived lymphocyte proliferation and suppression of B‐ or T‐lymphocyte‐mediated immune responses	[183]
In Vitro	Spleen and thymus lymphocytes (Wistar rats)	23–33 nm (TEM), 184 ± 16 nm (Average size, NTA)	Chitosan	↓ Μitogens‐induced splenic and thymic lymphocytes proliferative response	[184]
*In Ovo*	Broiler eggs from commercial breeder Ross 308 chickens (37 weeks old)	2–15 nm	‐	↓ TNF‐α, NF‐kB, and VEGF mRNA expression	[182]
In Vivo	Pupperfish (*Takifugu fasciatus*)	80 ± 15 nm (TEM), 141 ± 68 nm (NTA)	‐	↑ HSP70, HSP90, IgM and LZM	[179]
In Vivo	Common Carp (*Cyprinus carpio*)	<100 nm (BET)	‐	↑ Lysozyme activity, phagocytic activity, and IgM production	[180]
In Vivo	Trout (Juvenile rainbow trout)	<75 µm	‐	↓ TNF‐α, IL‐1β, IL‐10, Carbamoyl‐Phosphate Synthetase 2, Aspartate Transcarbamylase, and Dihydroorotase (CAD), superoxide dismutase (SOD) and Glutathione peroxidase (GPx) expression	[181]
In Vivo	Broilers strain (Cobb 500)	<100 nm	‐	↑ IgA, IgG, IgM and IL‐6 production	[185]
In Vivo	1‐d‐old Avian × Avian broiler chicks	95 ± 10 nm	Chitosan	↑ IgA, IgG, IgM production	[186]
In Vivo	Day‐old Ross308 chickens (♂)	5 nm (TEM)	PVP	↑ ESR, IL‐6, IgA, IgM, and IgY production	[187]
In Vivo	Skin UVB‐exposed mice	403 ± 10 nm (in PBS, DLS); 155 ± 46 nm (TEM)	Silica	↓ IL‐6 and IL‐1β production	[65]

## 5 Overview

In general, inorganic nanomaterials have an immunomodulatory effect that can either enhance or suppress immune responses depending on size, composition, and surface modifications (**Table** **5**).

**Table 5:** Key immune effects and mechanisms of immune modulation for AuNPs, AgNPs, and CuNPs.

Nanoparticle type	Key immune effects	Mechanisms of immune modulation
**AuNPs**	Dual role: both **pro‐inflammatory** and **immunosuppressive** effects; their effects varying based on size and coating.	‐ Activation of macrophages and DCs. ‐ Suppression of cytokine release. ‐ Modulation of ROS production.
**AgNPs**	Predominantly **pro‐inflammatory** responses but in certain sizes and with specific coatings, also exhibit **anti‐inflammatory** effects.	‐ Activation of NF‐κB pathway and cytokine release. ‐ Inhibition of inflammation via reduction of cytokine activity in certain sizes/coatings. ‐ Interaction with immune cell membranes causing oxidative stress.
**CuNPs**	Strong **immune‐modulatory** properties which include both i**mmunostimulant** and i**mmunosuppressive** effect.	‐ Stimulation of T‐cell and B‐cell proliferation in specific contexts. ‐ Inhibition of certain cytokines (e.g., IL‐6 or TNF‐α). ‐ Direct modulation of innate immune cell activation through oxidative and catalytic interactions. ‐Suppressing lymphocyte proliferation

In cultured cell models, metal nanoparticles interact directly with immune cells, such as macrophages, DCs, and T lymphocytes. These interactions can lead to various outcomes, ranging from pro‐inflammatory cytokine release to immunosuppressive effects. Their physicochemical characteristics influence the in vitro immune response to metal NPs. For example, smaller nanoparticles tend to exhibit higher reactivity due to increased surface area. The chemical nature of the metal can also induce different immune response, but there is a lack of specific investigations on this topic (**Table** **6**).

**Table 6:** Actual lacks and possible actions to improve the basic understanding of nano/immune interaction.

Lack	Reason	Possible Action
Comparative studies on the influence of physical, chemical, and physiological properties of metal‐based nanoparticles on the immune responses	Understand how size, shape, coating and composition of metal NPs influence the immunomodulation	Perform in deep investigations by focalizing on a single variable and employing standardized protocols
Standardized operating procedures in preclinical research.	Standardization of methods improve the reproducibility and comparability of results across different studies	Establish standard operative procedures (SOPs) to evaluate the nano/immune‐interaction (such as but not limited to: concentrations, incubation time, pre‐activation of in vitro models)
Influence of the biodistribution and clearance of metal nanoparticles on nano/immune‐interaction	The biokinetics of metal NPs varies with their intrinsic features (among which composition and surface decoration) and the administration pathway affecting the nano/immune‐interaction	Explore and compare the biodistribution and clearance mechanisms of metal NPs in immunocompetent and immunodeficient biomodels
Chronic exposure investigations on metal nanoparticles	Multiple or chronic exposure to metal NPs (and to the surface decoration) may lead to the depletion of immune cells or to autoimmune phenomena	Investigate multiple administrations and long‐term immunological effects of metal NPs administered by the mayor pathways (inhalation, injection, oral, and topical) in immunocompetent biomodels to analyze a potential development of an immune memory
Influence of protein corona formation on nano/immune‐interaction	Inorganic nanomaterials can acquire a new immunological identity once in the bloodstream, owing to protein corona formation	Analyze the composition of the protein corona on metal NPs in medium or in biological fluids during in vitro and in vivo experiments. Evaluate the effect on cells internalization and biokinetics
Spherical NPs only	The geometry of NPs impacts the protein corona formation and cells internalization	Evaluate the dependance of the nano/immune‐interaction on the geometry of NPs
Immune response associated to surface decoration	Immune response can be triggered by the surface functionalizing agents	Compare the immune modulation associated to the agents alone associated to NPs
Metal trace analysis in cells and tissues	The intracellular concentration of metals is associated to the type of cell as well as to the intrinsic features of NPs	Trace element quantification should be always supplied in nano/immune‐interaction evaluation to improve the consistency of experimental results

In in vivo studies, metal NPs show even more complex immunomodulatory effects due to the systemic nature of the immune response and the biodistribution of NPs across tissues. NPs administered to biomodels can be recognized by immune cells as foreign particles, leading to their uptake and subsequent activation of immune responses. Depending on the properties of the NPs, the immune response may be localized to specific tissues (such as the liver, spleen, or lungs) where macrophages are abundant, or it may become systemic. The immunomodulation associated with metal NPs in in vivo models is strongly connected to their systemic biodistribution and clearance. Even if the biodistribution of nanotherapeutics is determined from the administration strategy, non‐excretable metal NPs usually accumulate in organs such as the liver, spleen, lungs, and kidneys, where they may elicit localized immune responses.^[^ ^14^, ^188^ ^]^ Macrophages in these organs are known to phagocytize NPs, leading to either immune activation or, in some cases, immune depletion. Clearance of metal NPs from the body is another important factor in determining their immunological impact. NPs that are not efficiently cleared by the renal or hepatic pathways can persist in tissues, leading to chronic exposure and potential toxicity.^[^ ^8^, ^14^ ^]^ Indeed, the sustained presence of NPs may cause a prolonged inflammation or immune suppression leading to tissue damage, fibrosis, or impaired function (Table 6). Persistent metal NPs may also act as haptens, triggering adaptive immune responses such as the production of antibodies against the nanotherapeutic or its protein corona, which could interfere with future therapeutic applications or lead to hypersensitivity reactions. The development of an immune memory to metal NPs may either benefit immunotherapy (e.g., through enhanced targeting of tumors) or pose risks of autoimmunity if the organism recognizes self‐antigens associated with NPs‐treated cells. On the other hand, most of the reports analyze acute exposure to metal nanomaterials, and very little information has been collected regarding chronic exposure. Surface chemistry plays a critical role in determining the immunomodulatory effects of metal NPs. NPs can be functionalized with various biomolecules such as peptides and proteins, or with polymers to improve the biocompatibility, reduce cytotoxicity, and control immune responses. For example, PEGylation (decoration of nanoparticles with polyethylene glycol) has been widely used to evade the immediate immune recognition, delaying or reducing the immune activation.^[^ ^189^ ^]^ On the other hand, even if this feature may be advantageous to improve the accumulation to the target tissue, it can lead to unforeseen immune complications over prolonged exposure, such as developing immune tolerance or autoimmunity (Table 6). Additionally, immune reactions to the functionalizing agents, rather than the metal core, can trigger immune responses. A topic that has not been addressed in any of the analyzed works regards the formation of protein corona (both in vivo and in vitro). Indeed, immune cells can recognize the NPs indirectly through opsonization, i.e., serum proteins binding to the NPs surface, forming a protein corona that can lead to NPs clearance or immune activation regardless of the native surface functionalization. The dynamic and unpredictable nature of protein corona formation adds complexity to understanding how NPs interact with the immune system, sometimes leading to inconsistent results. On top of that, there are not standard operative procedures applied to preclinical research and the various reports focus on different aspects with field‐specific approaches, making the arena very difficult to navigate (Table 6). In this regard, some in vitro works report a pre‐activation of the immune cells with LPS before the NPs administration, changing the initial cellular status. Moreover, there is no agreement on the concentration of NPs with which the cells are incubated or the incubation timing. It is interesting to notice that almost all the works focus on spherical nanomaterials. Thus, it is actually impossible to discuss any potential dependence of the immune modulation to the shape of the metal NPs. Eventually, part of the inconsistency of the findings can be associated to the deficiency of data regarding the metal quantification in cells and tissues. Indeed, the intracellular concentration of metals can strongly vary because of the intrinsic features of the nanomaterials, causing a strong variation (even an opposite effect) on the overall immune stimulation. In this regard, investigations of nano/immune interactions should always be conducted with trace element quantification to avoid data misunderstanding (Table 6).

## 6 Conclusion and Perspectives

In summary, although comprehensive comparative research is still lacking, the analysis of the works presented in this review shows that: i) AuNPs tend to exhibit a dual role depending on size and coating, demonstrating both pro‐inflammatory and immunosuppressive effects; ii) AgNPs essentially provoke pro‐inflammatory responses but can also exhibit anti‐inflammatory effects at specific sizes and coatings; and iii) CuNPs display strong immune‐modulatory properties, enhancing inflammatory and adaptive immune responses in several contexts, while also suppressing lymphocyte proliferation or reducing certain cytokine levels (Table 5).

While the immunomodulatory effects of metal NPs offer exciting opportunities in oncology (and other biomedical fields), the challenges associated with their use in biological systems cannot be ignored (Table 6). Indeed, an uncontrolled inflammatory response poses significant concerns. Excessive inflammation can lead to cytotoxicity, impair cellular function, and even cause immunosuppression through mechanisms such as the overactivation of immune cells, leading to depletion. This is particularly concerning for therapeutic applications requiring precision to avoid damaging healthy cells. Thus, while the immunomodulatory effect of metal NPs holds promise for applications in clinical oncology, concerns regarding toxicity and unpredictable immune responses highlight the need for further research.

Investigating dose‐dependent effects, long‐term immunological impacts, and NPs opsonization with standardized protocols to evaluate the nano/immune‐interaction are essential topics for optimizing their safe and effective use in medical settings. Furthermore, cultured cell models often fail to account for the more complex in vivo immune network, which limits the predictive accuracy of these studies that should be validated in biomodels of increasing complexity. In vivo models reveal more complex outcomes due to the systemic biodistribution and clearance of the nanomaterials. While localized inflammation can occur at the site of accumulation, some studies indicate that chronic exposure to certain metal NPs may lead to immune suppression, mainly through the depletion of immune cells. Furthermore, prolonged administration may trigger adaptive immune responses, including antibody production, which should be investigated to avoid efficacy reduction in nanoparticle‐mediated therapies. Future exploration may focus on optimizing the nanoparticles’ design to fine‐tune immune responses to enhance the therapeutic efficacy while minimizing unwanted inflammatory or suppressive effects. This may involve engineering NPs with controlled release profiles, targeted delivery mechanisms, and surface modifications that precisely modulate immune cell engagement. Additionally, the long‐term immunological impacts of NPs exposure should be explored, particularly in relation to chronic diseases and immune disorders, where unintended immune modulation could have significant consequences. Beside nano/immune‐interaction, there is also the need to consider other translational challenges for noble metal NPs, among which are the potential long‐term metal persistence, standard and GLP/GMP production protocols, scaling up, and the involvement of clinicians from the early conceptualization stage (**Table** **7**).^[^ ^8^, ^190^ ^]^

**Table 7:** Translational challenges for noble metal nanotherapeutics.

Translational challenges
Standard protocols for the synthesis and characterization of NPs
SOPs for in vitro and in vivo experiments
Automated and scale‐up production
GLP/GMP protocols
High production costs
Involve clinicians from the early conceptualization stage

The integration of metal nanomaterials into cancer immunotherapy presents exciting opportunities for developing personalized and targeted treatments. By modulating immune responses and reducing TME‐mediated resistance, these strategies could transform oncology care, providing more effective and less toxic therapies for patients. However, while metal nanomaterials hold great promise in immunomodulatory applications, careful consideration of their immunological effects is essential. This review emphasizes that a deeper understanding of how different properties of the nanoparticles influence immune responses in both cultured cells and animal models will be crucial for developing safe and effective nanotechnologies for clinical use. We strongly believe that as research advances, metal nanomaterials will become a cornerstone of the future of personalized cancer therapy.

## Conflict of Interest

The authors declare no conflict of interest.

## Author Contributions

All Authors have discussed and contributed to writing the draft and the final version of the manuscript.
